# Mental rotation-related neural interactions between gender and cognitive strategy

**DOI:** 10.1162/imag_a_00310

**Published:** 2024-10-09

**Authors:** Nadia Marie Bersier, Sandra Arbula, Silvio Ionta, Raffaella I. Rumiati

**Affiliations:** Area of Neuroscience, Scuola Internazionale Superiore di Studi Avanzati, Trieste, Italy; Sensory-Motor Lab (SeMoLa), Department of Ophthalmology-University of Lausanne, Jules Gonin Eye Hospital-Fondation Asile des Aveugles, Lausanne, Switzerland; Department of Systems Medicine, University Rome ‘Tor Vergata’, Rome, Italy

**Keywords:** gender differences, mental rotation, fMRI, cognitive strategies, somatosensory processes

## Abstract

A long-standing history of research has focused on the differences between men and women in cognitive tasks, including that men would be more accurate and faster than women in mental rotation (MR). This advantage suggests that men would use an object-based cognitive strategy (OBS) to perform MR, whereas women would rely more on an effector-based cognitive strategy (EBS). To test this hypothesis, participants in the present study performed MR using OBS and EBS (plus a control condition) while their brain activity was recorded using fMRI. As sex hormones have often been reported to influence spatial ability, we also assessed the relationship between MR and testosterone levels and digit ratio. Behavioral results showed that (1) men performed faster MR than women in the OBS and control conditions, (2) men were more accurate than women in the OBS condition, and (3) women performed better in OBS than the other two conditions. No relationship was found between MR and testosterone or digit ratio. fMRI data showed that women in the OBS condition had greater activation than men in the inferior frontal and somatosensory cortices. Salivary testosterone levels had no effect on whole-brain activity. Combining behavioral and brain imaging data, these findings suggest that the additional somatosensory activation found in women during OBS somehow affects their MR, preventing the use of a purely spatial strategy and promoting the use of body-based sensorimotor processing, which would result in lower accuracy. These results support that gender differences in MR would be better explained by considering their relationship with the cognitive strategies used to perform MR.

## 1 Introduction

The role of gender in cognitive differences has attracted great interest for a long time, particularly with regard to spatial cognition. In this domain, mental rotation (MR) has often been found as easier/faster in men than in women (Halpern, 2013;Linn & Petersen, 1985;Shepard & Metzler, 1971;Voyer et al., 1995;Zapf et al., 2015). MR involves mentally rotating an object to understand its appearance after rotation without changing one’s viewpoint. Rather than being fixed, MR is sensitive to several variables, including both experimental factors such as the stimuli’s nature (Zeugin et al., 2020), point of view (Giovaola et al., 2022), and sensory modality (Pamplona et al., 2022), as well as contextual factors such as social interaction (Martinez et al., 2022). In this context, previous investigations on the potential influence of gender on MR have reported controversial results, indicating that gender might both primarily and secondarily affect MR (Guizzo et al., 2019;Jost & Jansen, 2023;Sanchis-Segura et al., 2018). The origin of such a controversy has been associated with several hypotheses, including that men and women would rely on different cognitive strategies.

For instance, it appears that the many strategies that can be used to solve MR tasks (e.g., mental rotation, perspective taking, counting cubes, local turns, and global shapes) vary in their reliance on holistic versus piecemeal processing (Hegarty, 2018) and that men would spontaneously use a holistic approach while women would prefer a piecemeal approach (Boone & Hegarty, 2017).

In particular, it has been proposed that MR can be accomplished by using an object-based strategy (OBS, taking the object itself as a reference frame) or an effector-based strategy (EBS, taking the viewer’s perspective as a reference frame) (Ionta et al., 2010;Zacks & Michelon, 2005). The use of OBS or EBS results in different psychometric patterns. Specifically, the Response Time (RT) for MR in OBS correlates with the orientation of the target image, regardless of any reference to the body of the participant. Conversely, in EBS the RTs for MR are affected by the participant’s body constraints in that, for instance, in EBS, the mental rotations oriented away from the midsagittal body plane (lateral rotations) take longer than rotations toward the midsagittal body plane (medial rotations) (Funk & Brugger, 2008;Pamplona et al., 2022). Since OBS has been associated with better accuracy and faster RTs (Halpern, 2013;Linn & Petersen, 1985;Shepard & Metzler, 1971) and men tend to show better performance when executing MR, it can be hypothesized that men spontaneously use OBS. Conversely, since women’s MR is slower and EBS is slower and more prone to errors, it might be the case that women spontaneously use EBS. Nevertheless, beyond spontaneous choices, MR strategies can be voluntarily adopted. For example, OBS can be imposed by telling participants: “Imagine that the figure is rotated on itself until it lines up with the other one” (Jordan, et al., 2002;Wolbers, 2003). However, EBS can be imposed by a command such as “Imagine that you turn the left figure with your hand until it matches the right figure” (Wolbers, 2003;Wraga et al., 2010).

Neuropsychological and imaging studies provided the necessary information about the neural correlates of MR. In particular, a meta-analysis (Tomasino & Gremese, 2016) found that, in addition to precentral and insular activations in the left hemisphere, MR bilaterally activates the inferior and superior parietal lobule, inferior frontal gyrus, middle frontal gyrus, supplementary motor area, inferior and middle occipital gyrus bilaterally, and cerebellum. In association with OBS, predominant activations are observed in occipital areas, as well as in right temporal and parietal regions. On top of the regions activated by OBS, EBS seems to activate mainly left sensorimotor regions.

Previous brain imaging studies reported very variable gender-related brain activations during MR. For instance, the inferior frontal cortex has been frequently associated with MR, but its activation has been reported as stronger in men than in women (Mendrek et al., 2011), stronger in women than in men (Hugdahl et al., 2006;Jiménez et al., 2010;Seurinck et al., 2004;Thomsen et al., 2000;Weiss et al., 2003), as well as equally (Semrud-Clikeman et al., 2012) and differently (Christova et al., 2008) strong in women and men. Similarly, the parietal cortex is considered one of the brain regions mainly activated by MR, but its activation has been found only in men (Hugdahl et al., 2006;Thomsen et al., 2000;Weiss et al., 2003), only in women (Jordan et al., 2002), and both in women and men (Semrud-Clikeman et al., 2012). Finally, the middle frontal cortex has been found as equally (Semrud-Clikeman et al., 2012) and differently (Christova et al., 2008) activated by MR in women and in men. Altogether, previous findings do not allow to draw definitive conclusions about the impact of gender on the brain activity patterns associated with MR.

Variability in genetics and hormone exposure that affects spatial aptitudes may also play a role in gender-related differences in MR. It is known that in rats, testicular hormones influence spatial abilities during the perinatal period. For instance, in male rats, the maze learning is adversely affected by neonatal castration (Dawson et al., 1975;Isgor & Sengelaub, 2003;Joseph et al., 1978;Williams et al., 1990), while in females, the maze performance is improved by neonatal testosterone therapy (Dawson et al., 1975;Isgor & Sengelaub, 1998;Joseph et al., 1978;Roof & Havens, 1992;Stewart et al., 1975). Similar interplays between hormones and spatial abilities have been observed in humans too (Puts et al., 2007). This notion is supported by the links between spatial skills and congenital adrenal hyperplasia. In this syndrome, an enzyme deficit diverts cortisol precursors to the androgen pathway, resulting in excessive production of adrenal androgens (Pang et al., 1980). While masculinized spatial abilities have been suggested in affected females (Hampson et al., 1998;Hines et al., 2003;Resnick et al., 1986), contrasting findings have also emerged (Helleday et al., 1994;Malouf et al., 2006;McGuire et al., 1975). For instance, some studies reported poorer spatial ability in congenital adrenal hyperplasia males than in healthy participants (Hampson et al., 1998;Hines et al., 2003), while others failed to report any significant differences (McGuire et al., 1975;Resnick et al., 1986). Another strand of literature suggests that the relationship between circulating testosterone and spatial abilities might not be linear, and would rather follow an inverted U-shaped curve, especially in men (Courvoisier et al., 2013;Moffat & Hampson, 1996;O’Connor et al., 2001). This model suggests that both low and high levels of testosterone could impair spatial cognition, while moderate levels might enhance it. The observed inverted U-shaped association may account for the observed discrepancy in findings across research on testosterone and spatial skills.

Investigations into potential links between early androgens and human spatial prowess have also sparked interest in digit ratio research. It has been suggested that the ratio of the second to fourth finger lengths (2D:4D) serves as a predictor of early androgen exposure, with men exhibiting a lower 2D:4D ratio than women (Manning et al., 1998). The sexual dimorphism in 2D:4D is believed to be impacted by prenatal sex hormones because of its early appearance. Numerous studies have, therefore, employed this anatomical marker to explore the potential impact of early androgens on spatial ability (Alexander, 2005;Austin et al., 2002;Coolican & Peters, 2003;Csatho et al., 2005;Kempel, Burk, et al., 2005;Kempel, Gohlke, et al., 2005;Loehlin et al., 2005;Manning & Taylor, 2001;McFadden & Shubel, 2003;Poulin et al., 2004;Putz et al., 2004). There is considerable variability in these outcomes, with some studies revealing positive associations, others uncovering negative correlations, and yet others detecting no significant connection, even within a single gender (review inPutz et al., 2004). Moreover, recent comprehensive research advises caution in interpreting digit ratios as proxies for testosterone levels. A meta-analysis bySorokowski and Kowal (2024), which included data from 8,077 participants, found no significant relationship between testosterone and the right or left 2D:4D, male or female 2D:4D, or between 2D:4D and testosterone measured in blood or saliva.

In the present study, we investigated the relationship between gender differences in MR-related performance (RTs and accuracy), cognitive strategy (limited to the comparison between OBS and EBS), and brain activity (fMRI), using an event-related design in fMRI. Our goal was to fill in the gaps in the field of gender differences in mental rotation by studying how men and women go about solving an MR task at the functional level, and how these activations vary according to the strategy used to solve the exercise. Thus, the aim of this study was to understand whether the use of a specific strategy could be at the root of gender differences in this domain. Furthermore, we tested whether different hormonal levels of testosterone were differentially associated with preferred strategies. We predicted that men would outperform women in the control and object-based conditions, whereas women’s performance would be affected by the triggered strategy. We also predicted that women would show more brain activity than men in sensorimotor regions.

## 2 Methods

### 2.1 Participants

Since education can influence MR (Moè et al., 2021), we uniformed our sample to only university students. Thus 65 participants were recruited via a local online platform. Two participants were excluded due to an interruption of the experiment, and one participant was excluded due to excessive head motion in the MRI scanner. This yielded a final sample of 63 participants (33 females, age 25.33 ± 3.8 years old, range 19–33; 30 males, age 26.2 ± 4.45 years old, range 20–35). Before entering the scanner, participants completed general health and fMRI compatibility questionnaires. Given the potential differences between perceived gender and biological sex (Wierenga et al., 2024), prior to the experiment all participants reported their perceived gender using a self-reported questionnaire. The questionnaire indicated that all participants had an alignment between perceived gender identity and biological sex. All participants were classified as right handed according to the Edinburgh Handedness Inventory (Oldfield, 1971). Any type of neurologic or psychiatric disorder, claustrophobia, any kind of ferromagnetic implant, and left handedness would have been considered an exclusion criterion for participation. Each participant gave written consent prior to their participation. The study was carried out in accordance with the 2013 Declaration of Helsinki criterion, with approval from the Friuli Venezia Giulia Regional Ethics Committee.

### 2.2 Procedure

Based on a previous behavioral study (Bersier et al., submitted), in the present event-related fMRI experiment, participants were shown 60 pairs of 3D figures. The two images of each pair corresponded to two out of the five figures that were taken from the dataset developed byShepard and Metzler (1971). For each pair, the first image represented a 3D figure rotated with respect to the vertical and was presented for 1 s tailed by a blank screen of a jittered duration (from 1 to 3 s). Based on the data recorded during a pilot phase of the study, all the figures had an intuitive vertical, defined mainly by the longer arms of the figure. During the pilot phase, we asked about 40 people to evaluate the verticality of 30 Shepard & Metzler figures. Then, only the figures that were rated as vertical by the majority of the evaluators were selected for the experiment. A total of 10 rotation angles were possible, ±30°, ±60°, ±90°, ±120°, and ±150°. Then, a cue appeared for 500 ms, representing either a circular arrow, a human hand, or a horizontal arrow and followed by a blank screen for 4.5 s. Then, the second image, representing a vertical 3D figure, was shown for 3 s. When the second image turned off, participants pressed one or another button to indicate whether the figure presented in the second image was congruent with the previously given instructions. To answer, participants had to press buttons on a keyboard placed on their chest. The trial ended with a jittered intertrial duration (6 s—duration of jitter 1), allowing all trials to have the same length of 15 s. The pairs of figures were presented in two blocks of 30 trials. Between the blocks, a short break allowed participants to rest. The set of 60 pairs was selected randomly from the larger set, with the restriction that all 5 figures and 10 rotation angles would be presented for each of them. All images were presented using PsychoPy (psychophysics software in Python (Peirce et al., 2019)) and delivered through MRI-compatible goggles attached to the head coil.

### 2.3 Conditions

**Fig. 1.**
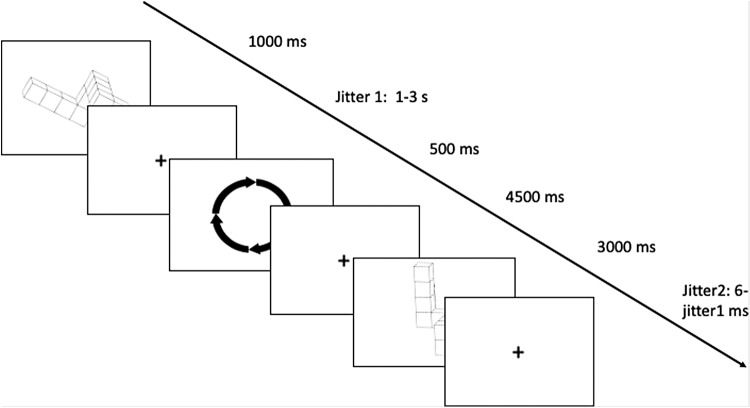
Example trial in the control condition. In this condition, subjects were asked to mentally rotate the first image in the picture-plane, in the direction given by the arrows until reaching the vertical position. When presented with the second figure, participants had to answer whether it was congruent or incongruent with the instruction given. In the example, the trial is congruent.

In the control condition (Fig. 1), the cue consisted of circular arrows, showing the direction of the rotation to be made (clockwise or counterclockwise in the picture-plane). The instructions were the following: “Look at the first figure, then the cue. Mentally rotate the first image in the direction indicated by the cue, until the figure is in its vertical position. Then compare the second figure appearing on the screen with the result of your mental rotation, and indicate whether it is the same or different.”

**Fig. 2.**
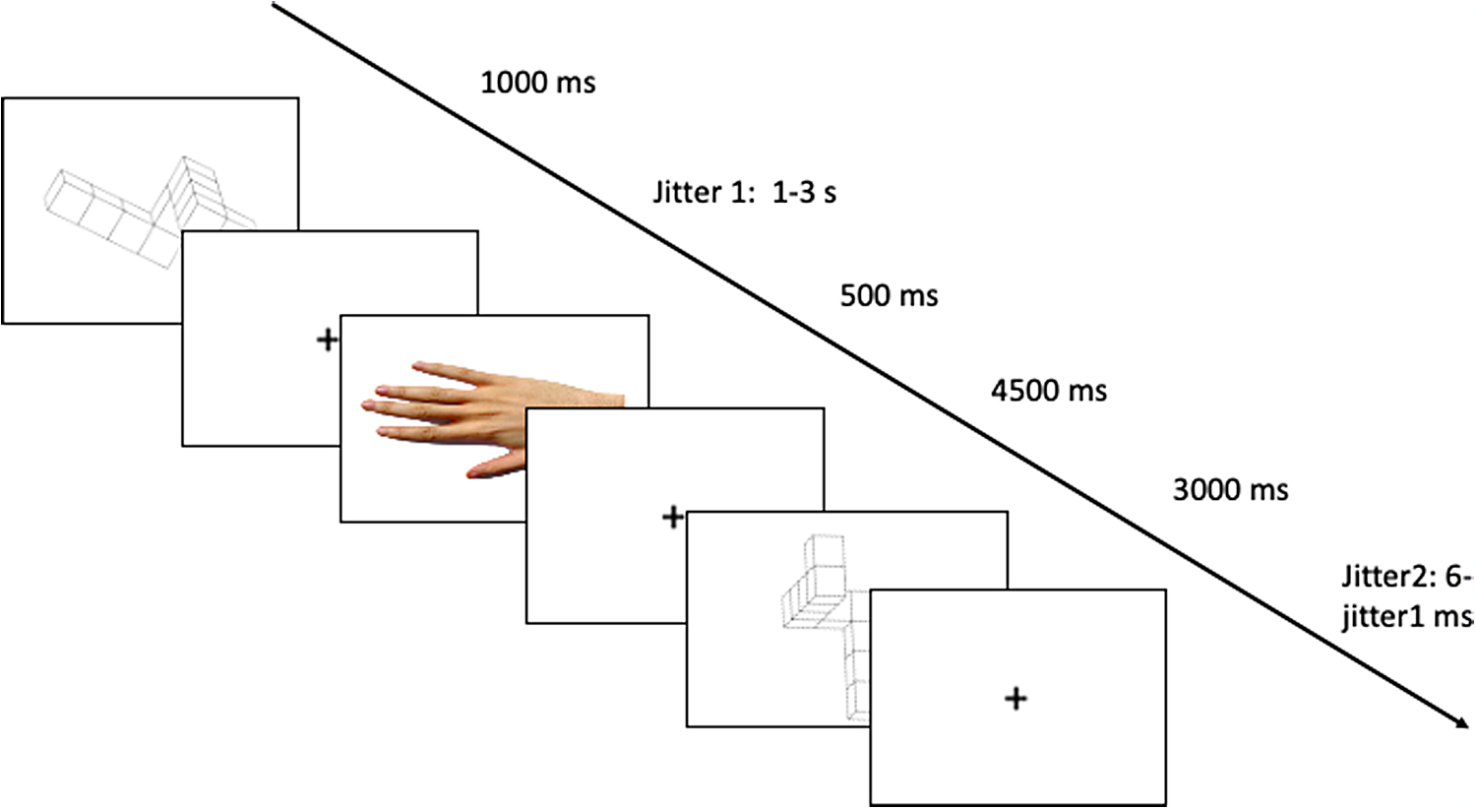
Example trial in the effector-based condition. Subjects were asked to mentally rotate the first image in the direction given by the hand in the picture-plane, until reaching the vertical position, following the natural movement of a human right hand. When presented with the second figure, participants had to answer whether it was congruent or incongruent with the instruction given. In the example, the trial is incongruent.

In the EBS condition (Fig. 2), the cue was replaced by a human right hand, pointing left or right. Participants were instructed to imagine positioning their own hand as shown by the cue, grasping the 3D figure shown at first, and mentally turning it in the picture-plane to its vertical position following the natural movement of the hand. Thus, if the hand cue pointed to the left, the movement to be imagined was clockwise, and if the hand pointed to the right, the imaged movement was counterclockwise. The explicit request to image using the right hand was intended to trigger an EBS and to use a reference frame the viewer’s perspective.

**Fig. 3.**
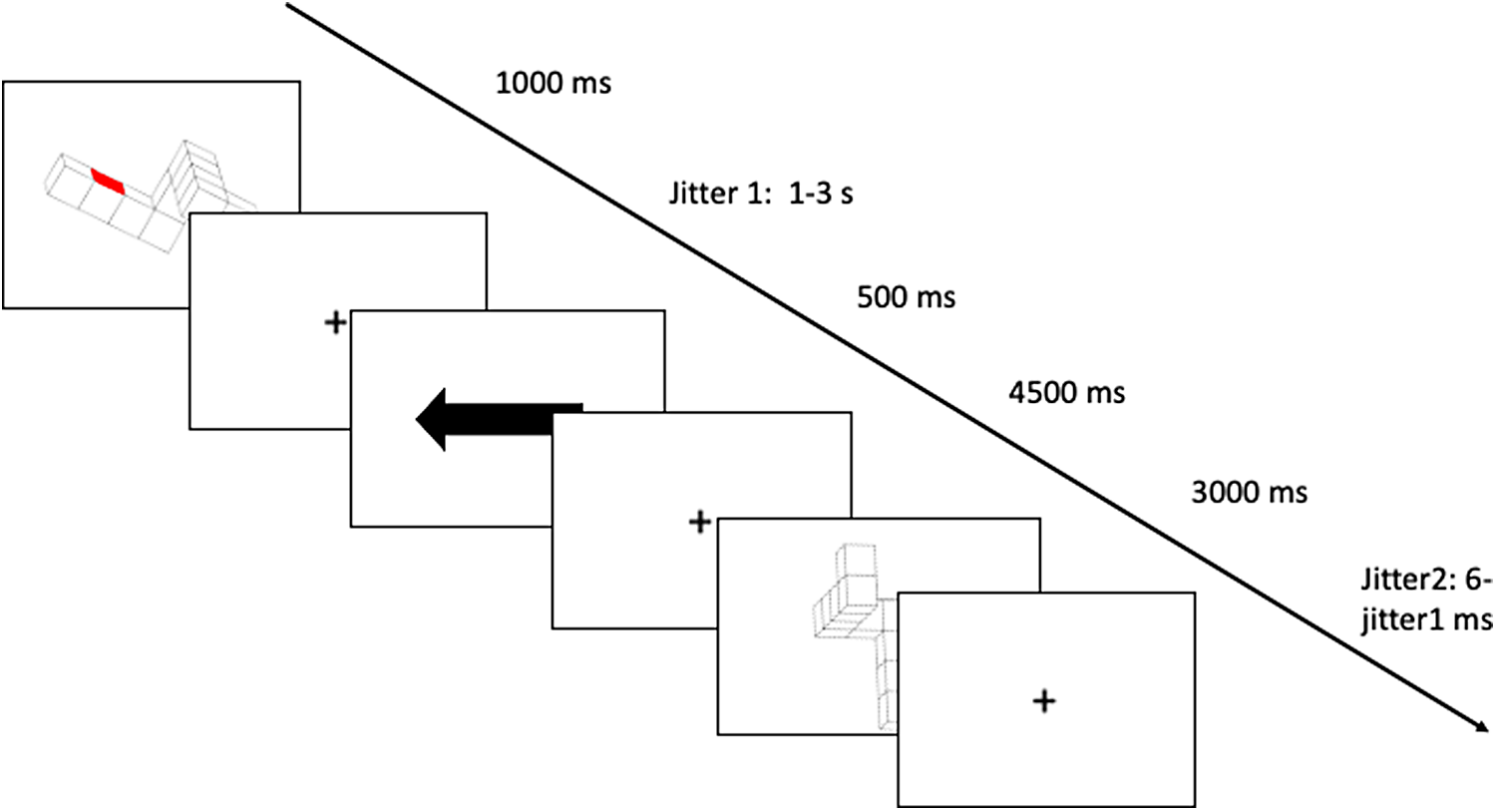
Example trial in the object-based condition. Subjects were asked to mentally rotate the first image in the picture-plane, and in such a way that the red mark would end up on the side indicated by the direction of the arrow. When presented with the second figure, participants had to answer whether it was congruent or incongruent with the instruction given. In the example, the trial is incongruent.

In the OBS condition (Fig. 3), the first figure had a red mark on one of its sides. The cue was a horizontal arrow, pointing either left or right. Participants had to mentally rotate the figure in the picture-plane until it reached the vertical, so that the red mark would be on the side indicated by the arrow. In this condition, the cue was no longer indicating the direction of rotation, but the side where the mark should be in the final position. On the second figure, the red mark was not shown. The red mark was intended to trigger a spatial (object-based) strategy, thus forcing the frame of reference to be the object itself.

In 50% of the cases, the answer was “congruent” (like inFig. 1), and in 50% of the cases, the answer was “incongruent.” To add some difficulty, the “incongruent” answers were divided into two categories: “mirrored” (like inFig. 2) or “wrong rotation” (like inFig. 3), to avoid memorization of the mirrored figures. All participants started the experiment with the control condition. Then, to account for the effect of the order of condition presentation, two orders were possible: order A in which the effector-based condition was presented before the object-based condition, and order B in which the object-based condition was presented before the effector-based condition. The experimental conditions were presented in a counterbalanced order. To familiarize with the task, for all 3 conditions, participants underwent a training session of 12 trials outside the scanner.

### 2.4 Hormone measure

Saliva was tested for free testosterone, which is a measure of the physiologically accessible portion of testosterone in the bloodstream (Rilling et al., 1996). Prior to entering the scanner, participants were requested to provide a sample, by salivating at least 2 mL into a plastic tube. Samples were then stored at -20 degrees, until transported to the analysis laboratory, where they were immediately analyzed. Testosterone levels were measured with an enzyme-linked immunosorbent assay (ELISA) protocol (manufactured by IBL International GmbH, Germany). The assay sensitivity was 29.8 pmol/. The average intra-assay coefficient of variation was 5.6% and interassay imprecision was 8.7%. Regarding prenatal testosterone, the fingertip to the middle of the basal crease on the hand’s palm was used to measure the lengths of the second (index finger) and fourth (ring) fingers. These numbers are divided to get the 2D:4D ratio (Brown et al., 2002).

### 2.5 MRI acquisition

The MRI acquisition and preprocessing used here closely followed the approach used inArbula et al. (2021). For consistency, with the editor’s permission, we reproduce the text used to describe it here, noting differences as appropriate.

MRI data were collected using a 3 Tesla whole-body scanner (Achieva Philips) with an 8-channel head coil at the “S. Maria della Misericordia” hospital in Udine. For each of the six runs of the mental rotation task, we collected 231 functional image volumes comprising 37 continuous axial slices. These images were acquired using a T2*-weighted echo-planar sequence with the following parameters: repetition time (TR) of 2 s, echo time (TE) of 30 ms, flip angle (FA) of 82 degrees, voxel size of 3 × 3 × 3 mm, and acquisition matrix of 80 × 80. At the beginning of the session, we acquired a high-resolution T1-weighted anatomical image consisting of 170 sagittal slices. The parameters for this image were as follows: TR/TE of 8.1/3.7 ms, FA of 12 degrees, voxel size of 1 × 1 × 1 mm, and acquisition matrix of 240 × 240. To account for spatial distortion in the functional images, we acquired a pair of spin-echo images with opposite phase encoding directions, matching the orientation of the functional scans. These spin-echo images were obtained at the beginning of each condition, for a total of three sequences.

### 2.6 MRI quality assessment process

MRI data were converted using the Dcm2Bids program (https://github.com/cbedetti/Dcm2Bids) from DICOM format to the Brain Imaging Data Structure (BIDS;https://bids.neuroimaging.io/). The MRI Quality Control tool (MRIQC) (Esteban et al., 2017) was then used to assess the quality of the structural and functional data. Using the MRIQCeption tool (https://github.com/elizabethbeard/mriqception), a comparison was done between the acquired quality metrics and a set of metrics from the MRIQC online API (Esteban et al., 2019).

### 2.7 MRI preprocessing

The fMRI data were preprocessed using fMRIPrep version 1.5.1rc2 ((Esteban et al., 2019); RRID:SCR_016216) a Nipype ((Gorgolewski et al., 2011) RRID:SCR_002502)-based tool. The T1-weighted (T1w) volumes underwent intensity nonuniformity correction (INU) using N4BiasFieldCorrection (Tustison et al., 2010) distributed with ANTs 2.3.3 ((Avants et al., 2008) RRID:SCR_004757) and used as T1w-reference throughout the workflow. They were then skull stripped using antsBrainExtraction.sh v2.2.0, based on the OASIS template. Brain surfaces were reconstructed using recon-all from FreeSurfer v6.0.1 ((Dale et al., 1999) RRID:SCR_001847), and the brain mask obtained previously was further refined with a customized approach reconciling ANTs-derived and FreeSurfer-derived segmentations of the cortical gray matter from Mindboggle ((Klein et al., 2017); RRID:SCR_002438). To achieve spatial normalization, the data were registered to the ICBM 152 Nonlinear Asymmetrical template version 2009c ((Fonov et al., 2009); RRID:SCR_008796) using the antsRegistration tool of ANTs v2.2.0 ((Avants et al., 2008); RRID:SCR_004757). This registration involved brain-extracted versions of both the T1w volume and the template. Subsequently, brain tissue segmentation for cerebrospinal fluid (CSF), white matter (WM), and gray matter (GM) was performed on the brain-extracted T1w data using fast (FSL v5.0.9); ((Zhang et al., 2001); RRID:SCR_002823). The functional data underwent slice time correction using 3dTshift from AFNI v16.2.07 ((Cox, 1996); RRID:SCR_005927) and motion correction using mcflirt (FSL v5.0.9) (Jenkinson et al., 2002). Distortion correction was performed using an implementation of the TOPUP technique (Andersson et al., 2003) with 3dQwarp (AFNI v16.2.07); (Cox, 1996). Coregistration to the corresponding T1w data was done using boundary-based registration (Greve & Fischl, 2009) with six degrees of freedom, employing bbregister (FreeSurfer v6.0.1). The transformations for motion correction, field distortion correction warp, BOLD-to-T1w transformation, and T1w-to-template (MNI) warp were combined and applied in a single step using antsApplyTransforms (ANTs v2.2.0) with Lanczos interpolation.

To handle physiological noise, CompCor (Behzadi et al., 2007) was used to extract principal components for the anatomical CompCor variants (aCompCor). A mask excluding cortical signal was created by eroding the brain mask, leaving only subcortical structures. Six aCompCor components were calculated within the intersection of the subcortical mask and the union of CSF and WM masks derived from the T1w data, projected to the native space of each functional run. Frame-wise displacement (FD) and DVARS (Power et al., 2014) were calculated for each functional run using the Nipype implementations.

Further processing involved masking the functional data using the brain mask obtained from fMRIPrep. Fourteen fMRIPrep-derived confounds (six motion parameters, FD, standardized DVARS, and six aCompCor components) were removed at a voxel-wise level using the Denoiser tool (Tustison et al., 2010). Finally, the functional data were spatially smoothed using a Gaussian kernel with a full-width at half-maximum of 6 mm.

### 2.8 Behavioral data analysis

We collected accuracy and RTs data. RTs were filtered for errors and outliers above 3 standard deviations from the group mean for each condition. Statistical analyses were performed with RStudio (https://rstudio.com/). Analyses of variance (ANOVA) were performed to explain the mean reaction time as well as accuracy, with gender, condition, testosterone, digit ratio, and order of the task as factors. Our main hypotheses of gender differences in MR strategies were additionally tested by contrasting condition|gender and gender|condition (library “emmeans”) for the planned comparisons as specified in the Introduction section. If a significant effect was found for expected differences (such as differences in favor of males for accuracy and reaction time), paired comparisons Tukey-corrected for multiple comparisons were made.

Regarding testosterone and digit ratios, additional correlations were performed with both accuracy and reaction time. Since men have significantly higher levels of free testosterone than women, these analyses were carried out separately for the two groups.

### 2.9 fMRI data analyses

#### 2.9.1 First-level GLM analysis

For the first-level General Linear Model (GLM) analysis, FSL FEAT was used (www.fmrib.ox.ac.uk/fsl). A separate GLM model was constructed for each participant and each run, where the three experimental conditions (control, object-based, and effector-based) served as the regressors of interest, and their temporal derivatives were included as regressors of no interest. The regressors were convolved with a double-gamma hemodynamic response function and timed with the presentation of the first image. The period of interest for the hemodynamic response analysis is described in the section Univariate analysis. We used FILM prewhitening to adjust for autocorrelation, and a high-pass filter with a 100-s cutoff was used to remove low-frequency drifts.

#### 2.9.2 Group-level GLM analysis

Using mixed effects (FLAME 1) as implemented in FSL, a whole-brain group-level analysis was carried out to determine mean group effects. A cluster-based method was used to threshold the statistical map, using a*Z*> 2.3 threshold. Family-wise error correction was then applied to make adjustments at*p*= .05.

#### 2.9.3 Univariate analysis

To study precisely the time windows during which the mental rotation was performed, we isolated the period of interest as the one following the cue, for a duration of 4.5 s. To explore the distinctive neural activations corresponding to each condition, six separate contrasts were performed. Additionally, we included group as a regressor to determine whether any observed effects were influenced by the gender.

### 2.10 Testosterone analysis

Using within-group linear regression analysis, correlations between salivary testosterone and mental rotation-related brain activity were examined to look into a possible relationship with sex hormones. Testosterone levels were mean centered across all subjects and included as a regressor in the third level analysis GLM model. The analysis was done for all individuals included in the same group, but given that men naturally have more testosterone than women, the analysis was spotted for both groups separately. The analysis was repeated for each experimental condition separately. Additionally, to investigate a potential nonlinear relationship between free testosterone and performance (accuracy and reaction time), we ran quadratic regressions to verify that a possible U-shaped relationship did not exist.

In whole-brain and region-of-interest (ROI—described below) analyses, we investigated whether correlations between brain activations and testosterone levels could be found. The ROI analysis minimizes the amount of voxel-wise multiple comparisons as compared with whole-brain mapping and may identify effects that would go unnoticed otherwise. We selected the difficult (150 degrees of rotation) and easy trials (30 degrees of rotation) to create a contrast representing the maximal cognitive effort. Runs were then averaged across conditions and beta values for each subject extracted and correlated (Pearson correlation) to their respective salivary testosterone. Regions of interest (ROIs) for visuospatial strategy and motor strategy were chosen according toTomasino and Gremese (2016)meta-analysis. Regions specifically associated for visuospatial strategy were (i) R Precuneus, (ii) R Superior frontal gyrus, (iii) R Superior occipital gyrus, (iv) L Middle occipital gyrus, (v) L Superior parietal lobe, (vi) L Inferior temporal gyrus, (vii) L Middle occipital gyrus, and (viii) R Posterior medial frontal gyrus. Regions specifically associated for motor strategy (i) R Postcentral gyrus (Areas 2, 3b, 4p), (ii) L Inferior parietal lobe, postcentral gyrus (Areas 2, 1, 3b), (iii) L Superior parietal lobe, and (iv) R Angular gyrus. ROIs were created by designing 5-mm-radius spheres centered in the coordinates. This procedure was done across all conditions, to check for differences in strategies.

## 3 Results

### 3.1 Behavioral results

**Fig. 4.**
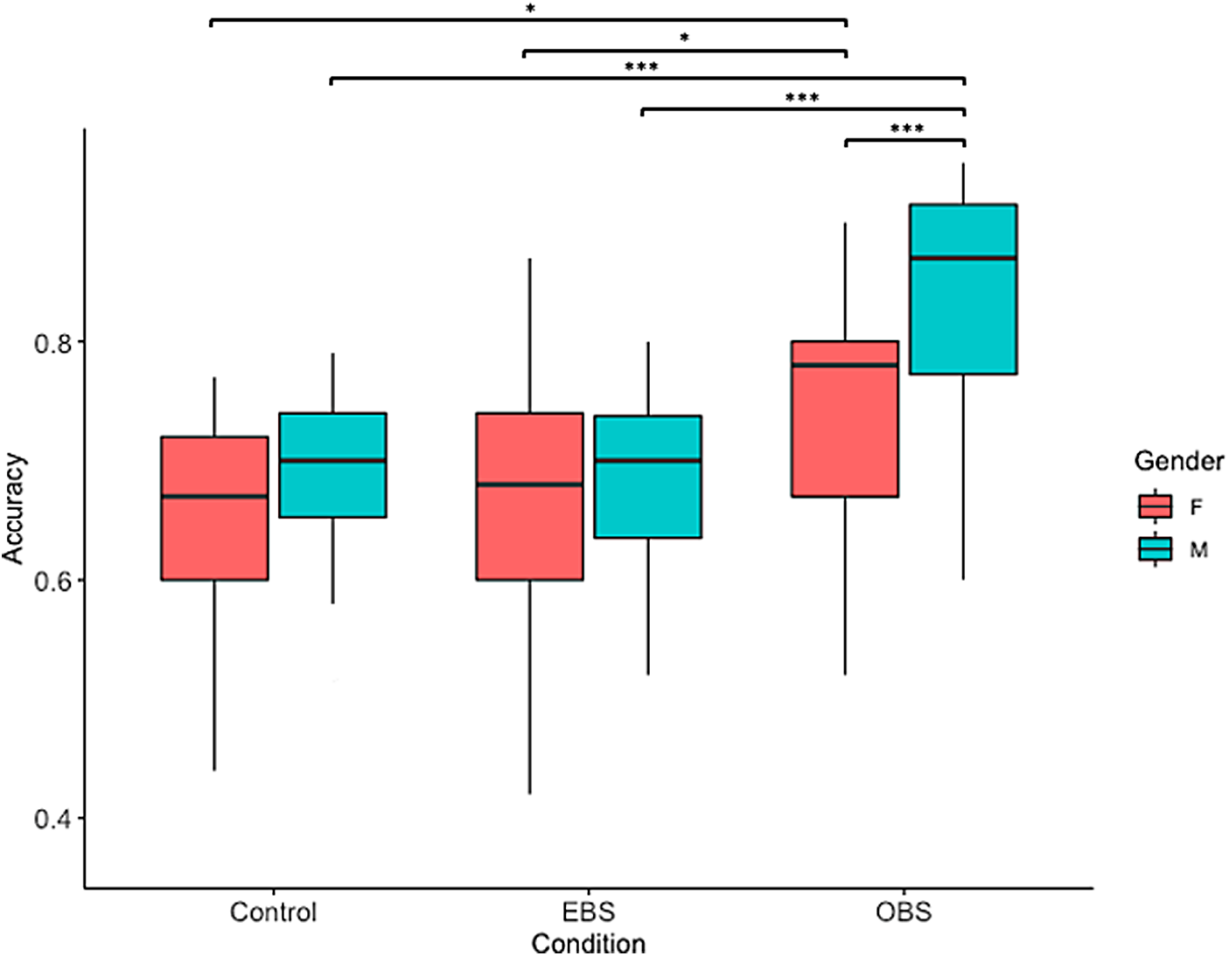
Accuracy across conditions and separated by gender. Paired comparisons with*t*-tests (corrected for Tukey) revealed significant differences within the OBS condition. For men, paired comparisons with*t*-tests (corrected for Tukey) revealed significant differences between the OBS and the EBS conditions, as well as between the OBS and EBS conditions. Similarly for women, significant differences were found between the OBS and control conditions, as well as between OBS and EBS conditions. *denotes a significant difference p < 0.05, ***denotes a significant difference p < 0.001.

The analysis of variance revealed significant main effects of condition [*F*(2, 180) = 18.19,*p*< .001] and gender [*F*(1, 180) = 8.66,*p*= .003] on accuracy. Additionally, a significant interaction between the condition and the order of the tasks was found [*F*(2, 180) = 3.84,*p*= .023]. When looking at pairwise differences between genders within each condition, we found a significant difference among women between the OBS and control conditions [*t*(1, 180) = -3.04,*p*= .007], as well as between the OBS and EBS [*t*(1, 180) = -2.68,*p*= .021]. When doing the same among men, the same significant differences were found, between OBS and control conditions [*t*(1, 180) = -4.78,*p*< .001], and OBS and EBS [*t*(1, 180) = -4.64,*p*< .001] (Fig. 4).

When investigating the main effect of gender, the only significant difference was found in the OBS, where men outperformed women [*t*(1, 180) = -2.99,*p*= .003] (Fig. 4,Table 1).

**Table 1.** Mean accuracy and reaction time (RT; seconds) for each condition across groups.

	Accuracy Mean ± SD			RT(s) Mean ± SD		
Condition	Control	OBS	EBS	Control	OBS	EBS
Men	0.68 ± 0.09	0.81 ± 0.14	0.68 ± 0.06	1.14 ± 0.32	1.05 ± 0.31	1.08 ± 0.33
Women	0.65 ± 0.08	0.73 ± 0.13	0.66 ± 0.11	1.34 ± 0.36	1.27 ± 0.38	1.23 ± 0.41

**Fig. 5.**
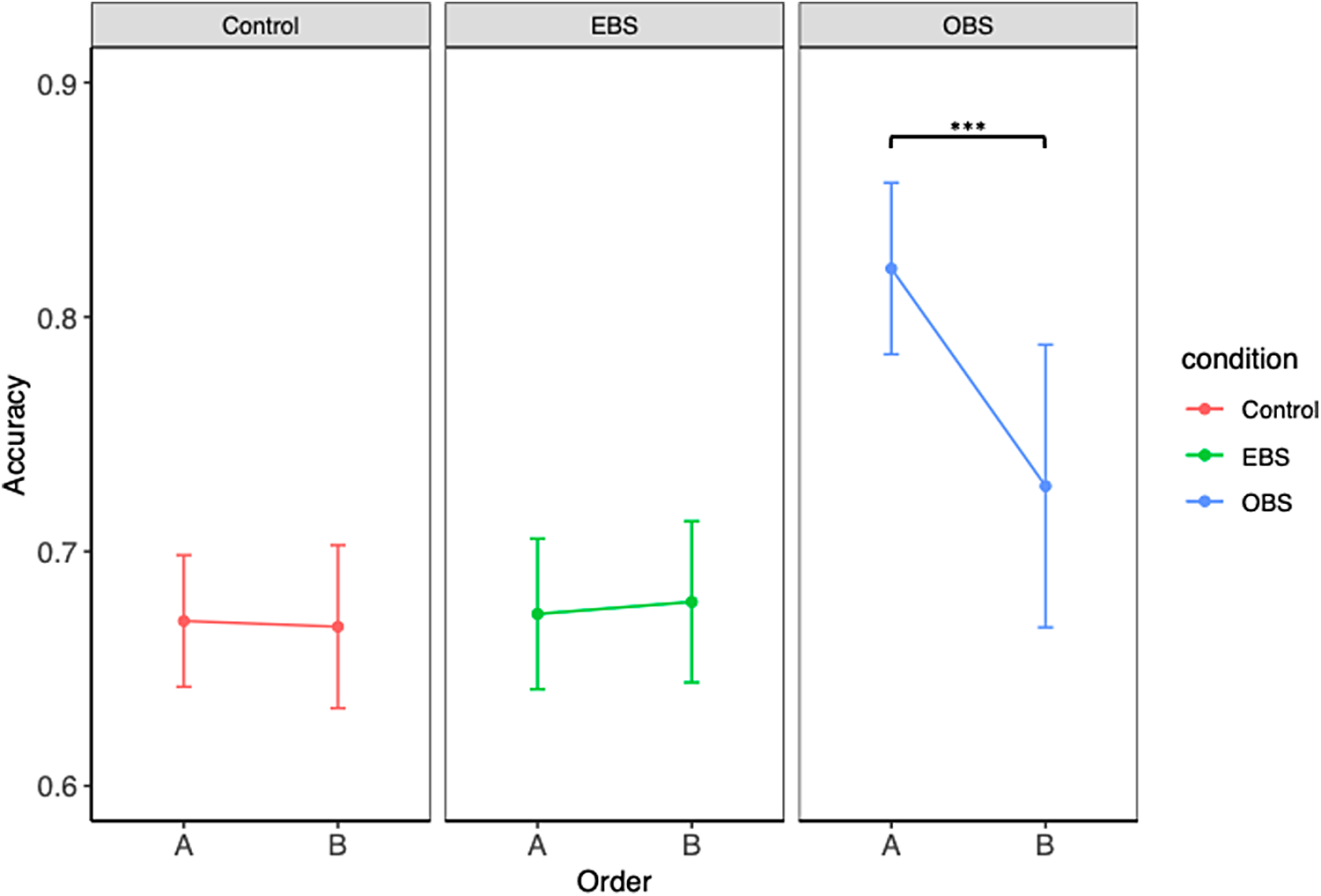
Accuracy across conditions and separated by order.*t*-Tests corrected for multiple comparisons revealed a significant difference within the OBS condition where the scores for the A order were higher than for the B order,*t*(1, 180) = 3.29,*p*= .001. ***denotes a significant difference p < 0.001.

Regarding the interaction between condition and the order of the tasks, a significant effect was found in the OBS condition [*t*(1, 180) = 3.29,*p*= .001)], where scores were higher in the A order of the experiment (in which the effector-based condition was presented before the object-based condition) than in the B order (Fig. 5).

**Fig. 6.**
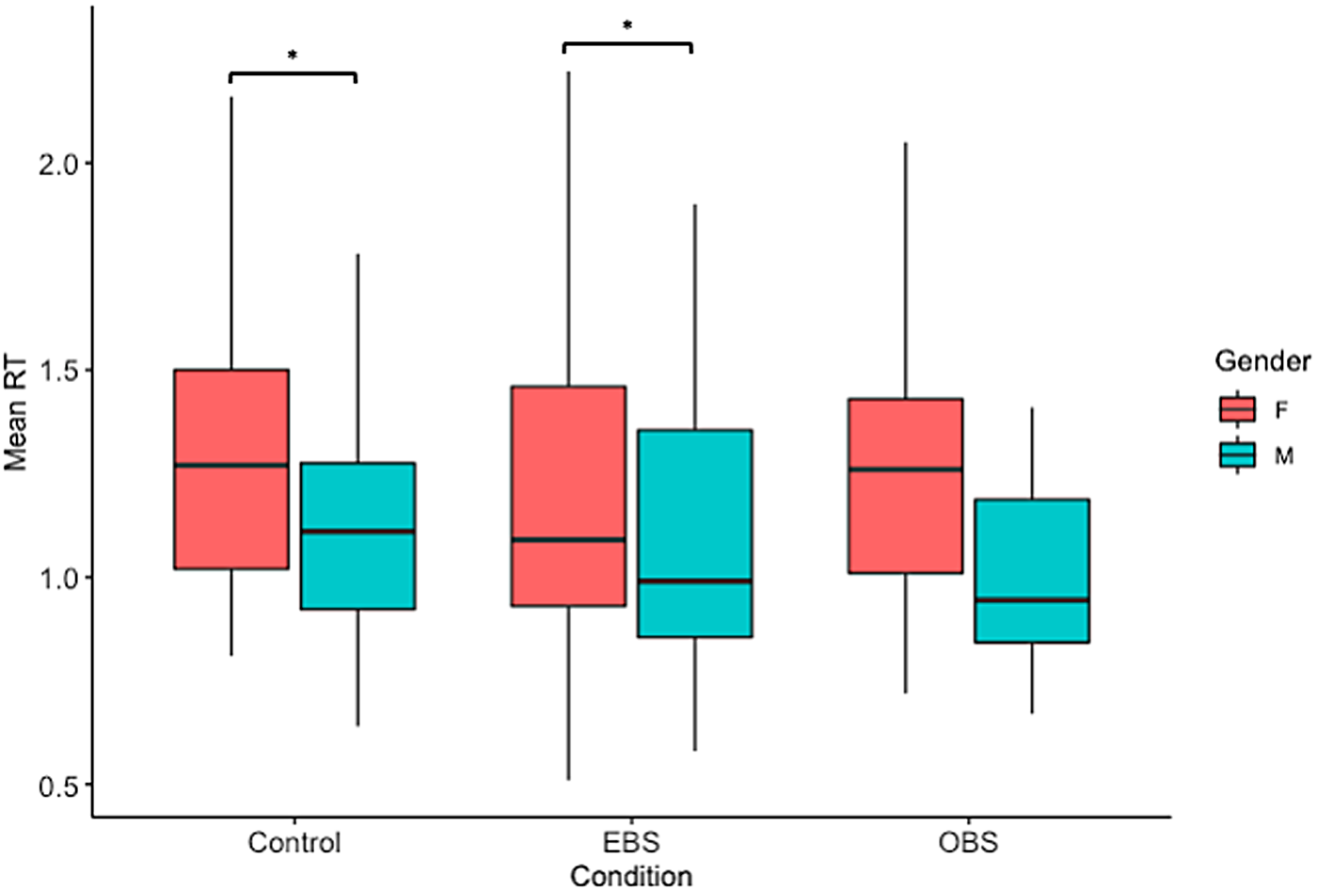
Mean reaction time for each condition separated by gender. Paired comparisons with*t*-tests (corrected for Tukey) revealed significant differences within the OBS condition and the control condition. *denotes a significant difference p < 0.05.

As for reaction times, the analysis of variance revealed a significant effect of gender. Planned comparisons showed that men had shorter reaction time than women in the OBS [*t*(1, 183) = 2.451,*p*= .0152], as well as in the control condition [*t*(1, 183) = 2.234,*p*= .0267]. No gender difference was revealed within the EBS (Fig. 6).

### 3.2 Testosterone results

The male group presented a mean free testosterone concentration of 257 pmol/L (SD = 134), while the female group presented a mean concentration of 59.3 pmol/L (SD = 35.3). For the digit ratio, men had a ratio of 0.97 (SD = 0.04) between the second and fourth digit, while women had a ratio of 0.98 (SD = 0.03). No link was found between measures of testosterone and behavioral data. Salivary testosterone as well as digit ratio showed no significant correlation with accuracy nor RT with the general MR score, or with any of the specific conditions (Table 2). When inserted as a factor in the analysis of variance, separately for men and women, no significant effect arose. Quadratic regressions to check for a potential U-shaped link between performance (accuracy and reaction time) and salivary testosterone also failed to produce significant results.

**Table 2.** Correlation (Pearson’s*r*) table for testosterone measures and between group performance across conditions as well as for the mean MR accuracy and reaction times.

	Men				Women			
	*Free testosterone*		*Digit Ratio*		*Free testosterone*		*Digit Ratio*	
	*r* accuracy	*r* RT	*r* accuracy	*r* RT	*r* accuracy	*r* RT	*r* accuracy	*r* RT
Mean	0.14	-0.06	-0.08	-0.02	0.04	0.12	-0.06	-0.02
Control	0.15	-0.07	-0.001	-0.07	-0.08	0.13	-0.13	-0.07
OBS	0.11	-0.04	0.02	-0.02	-0.21	0.28	0.02	0.16
EBS	0.16	-0.06	-0.18	-0.03	0.27	-0.09	-0.11	0.007

### 3.3 Neuroimaging results

#### 3.3.1 Whole brain

**Fig. 7.**
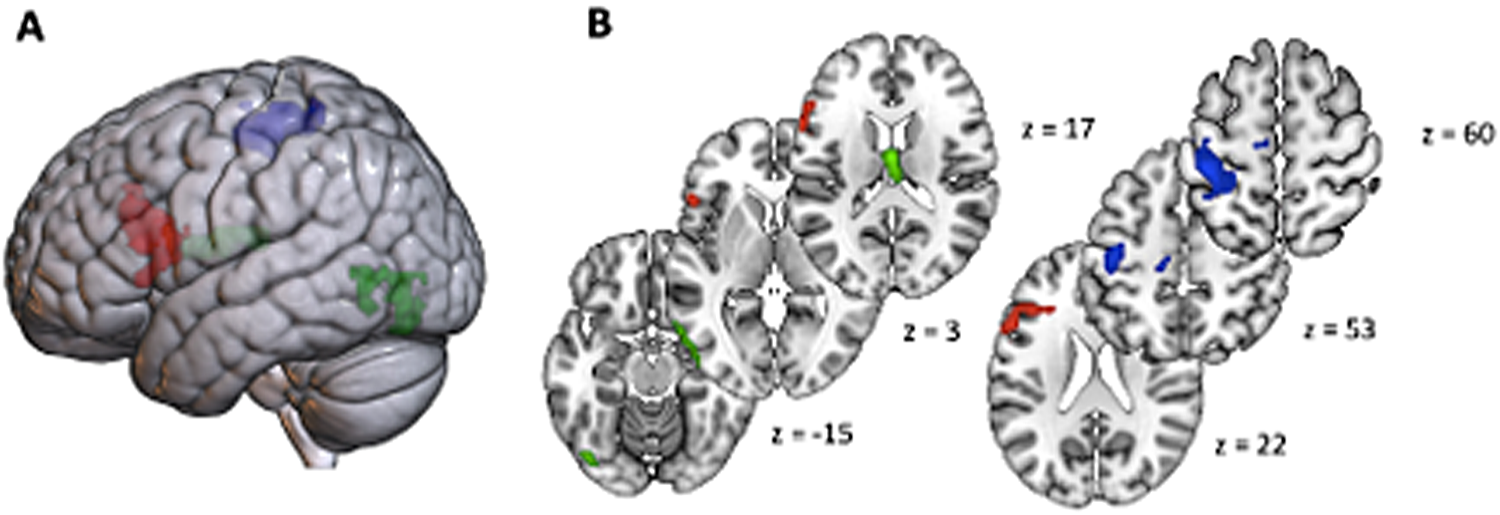
Rendered 3D brain (A) and axial slices (B) showing significant contrast activations. Brain activations from the whole-brain voxel-wise OBS–control contrast are shown in red; brain activations from EBS–OBS contrast are shown in blue; brain activations from EBS–control are shown in green. The statistical maps were assessed with a cluster-based threshold of*Z*> 2.3, corrected at*p*= .05 (family-wise error correction).

The OBS–control contrast at the group level showed a main cluster of activation in the left inferior frontal cortex, in the opercular and triangular sections (Fig. 7;Table 3).

**Table 3.** Significant clusters from whole brain.

	MNI			Peak Z	Cluster level	
	x	y	z		Size	*p*
EFFECTOR-BASED vs. OBJECT-BASED						
L Postcentral gyrus	-39.5	-30.5	65.5	3.67	163	.000531
L Precentral gyrus	-39.6	-15.5	53.5	3.54		
L Mid cingulum (BA6)	-12.5	-24.5	50.5	3.42		
L Postcentral gyrus	-27.5	-39.5	59.5	3.26		
L Superior parietal lobe	-33.5	-45.5	65.5	3.13		
L Supplementary motor	-9.5	-18.5	59.5	2.75		

EFFECTOR-BASED vs. CONTROL						
L Thalamus	-0.5	-9.5	17.5	4.45	89	.00229
R Caudate	8.5	5.5	11.5	2.99		
L Caudate	-6.5	2.5	11.5	2.87		
R Thalamus	11.5	-12.5	11.5	2.52		
L Middle temporal	-60.5	-63.5	-0.5	4.54	85	.0294
L Inferior occipital	-48.5	-78.5	-9.5	3.52		
L Fusiform gyrus	-42.5	-78.5	-15.5	3.37		
L Middle temporal	-57.5	-69.5	2.5	3.25		
L Inferior temporal	-54.5	-63.5	-9.5	2.98		
L Middle occipital	-48.5	-75.5	-0.5	2.74		

OBJECT-BASED vs. CONTROL						
L inferior frontal gyrus, opercular part	-57.5	23.5	14.5	3.5	164	.000391
L Inferior frontal gyrus, opercular part	-48.5	26.5	29.5	3.46		
L Inferior frontal gyrus, triangular part	-51	20.5	2.5	3.33		
L Inferior frontal gyrus, triangular part	-36.5	26.5	32.5	3.26		
L Inferior frontal gyrus, triangular part	-30.5	26.5	23.5	3.2		
L Inferior frontal gyrus, opercular part	-57.5	11.5	8.5	3.13		

F > M for OBJECT-BASED vs. CONTROL						
R Superior temporal	62.5	-27.5	20.5	3.81	110	.00651
R Rolandic operculum	53.5	-15.5	14.5	3.79		
R Supramarginal gyrus	50.5	-27.5	32.5	3.47		
R Superior temporal	62.5	-21.5	17.5	3.17		
R Inferior frontal gyrus, triangular part	56.5	21.5	2.5	2.53		

The EBS–OBS content contrast at the group level showed left activations in regions typically associated with motor and sensorimotor processing, comprising the supplementary motor cortex, as well as postcentral and precentral gyrus, the middle cingulum, and the superior parietal lobe (Fig. 7;Table 3).

**Fig. 8.**
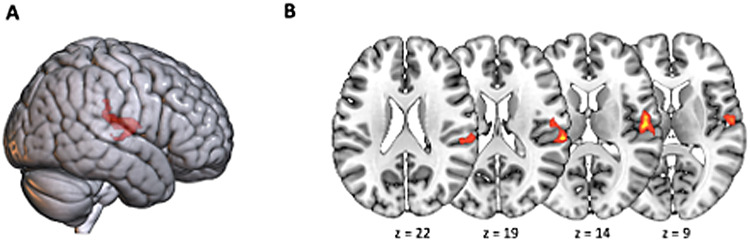
Rendered 3D brain (A) and axial slices (B) showing significant OBS–control contrast activations with gender as a covariate. Brain activations from the whole-brain voxel-wise OBS–control contrast for F > M. The statistical map was assessed with a cluster-based threshold of*Z*> 2.3, corrected at*p*= .05 (family-wise error correction).

The EBS–control contrast at the group level showed two main clusters of activations. The first comprised the thalamus and caudate, bilaterally. The second comprised exclusively left areas and included the middle temporal, inferior, and middle occipital cortex, as well as fusiform gyrus (Fig. 7;Table 3).

Finally, when doing a two-sample*t*-test to study the effect of gender, a significant cluster arose in the F > M contrast. Areas were located in the right hemisphere and englobed the superior temporal lobe, the rolandi operculum, the supramarginal gyrus, and the inferior frontal gyrus.

#### 3.3.2 Testosterone results

When included in the GLM as a regressor, levels of salivary testosterone showed no interaction with whole-brain activation, for any of the experimental conditions. ROI analyses for the hard < easy contrast were conducted with the areas cited in Section 2.7.4 and corrected for multiple comparisons. Individual beta-maps within the ROIs were correlated with the testosterone levels for each participant and each condition. Correlation tests did not reveal any significant correlation between the activations within the ROIs and the individual salivary testosterone levels.

## 4 Discussion

The present study investigated how the brain activity related to MR is affected by cognitive strategy and gender. The results showed that (1) men’s and women’s behavioral performance was affected by the cognitive strategy used to solve the MR task (higher accuracy in the OBS condition for men than for women), (2) imposing different strategies to perform MR activated different brain regions, and (3) gender differences were reflected in different brain activation patterns for MR.

### 4.1 Cognitive strategy and brain activity

The OBS significantly activated the pars opercularis with respect to the control condition. Some studies have shown that this area is activated during spatial processing tasks, such as MR and space-related processing tasks (Wiesen et al., 2022). In particular, the left pars opercularis has been found to be more active during mental rotation of objects and images (Tomasino & Gremese, 2016). In line with these findings, it seems that individuals successfully engage into a “spatial” strategy in comparison with the control condition. Moreover, the object-based condition showed greater accuracy for both males and females at the behavioral level, compared with the control condition. Thus, activation of the pars opercularis in OBS engagement is associated with greater overall accuracy and suggests that it is a key structure in explaining the spatial strategy efficiency.

In the EBS–OBS contrast, significant activation of the motor and sensorimotor cortices was observed. In EBS, participants were specifically guided to mentally reach, grasp, and rotate the figure using their right hand. The imagery results seem to reflect the use of a motor strategy, as well as embodiment processes, indicating that participants were successfully triggered into an effector-based perspective, with respect to the OBS condition. Interestingly, at the behavioral level, MR in OBS had greater accuracy than in EBS and control conditions (seeFig. 4). The novelty here lies in the fact that we were able to link performance, strategy, and brain activation, and thus provide a clue as to why motor strategy is commonly less effective than others. It is, therefore, possible that the additional activation of sensorimotor regions may compromise the efficiency of MR processes, resulting in poorer performance.

With respect to the control condition, EBS activated the thalamus. Although it is not the brain region the most prominently associated with MR, the thalamus has been involved in MR-related research (Butler et al., 2006;Calhoun et al., 2001;Potvin et al., 2013;Schweinsburg et al., 2012;Thérien et al., 2022). In addition to being a relay for most sensory information, this structure also has a key role in the body representation system, which encompasses the neuronal representations of the body and is, therefore, crucial for understanding how motor functions operate (Ehrsson et al., 2003;Longo & Haggard, 2010). In a series of studies,Naito and Ehrsson (2006)andNaito et al. (1999,^2002^,^2016^) suggested that the thalamus is a component of the sensorimotor control network that helps with both online movement control and the development of body images. Recently, it has also been shown that the thalamus plays an important role in vestibular processes (review inWijesinghe et al., 2015). Therefore, the thalamic activation found in our contrast might be related to the self-oriented and vestibular processing induced using an effector-based strategy in that condition. Finally, EBS significantly activated the occipitotemporal cortex. This area is known to be involved in the visual-spatial processing of the rotated objects and plays a crucial role in the mental manipulation of visual information (Haxby et al., 2001). Hence, the observation of its activation in a context eliciting manual object manipulation is highly coherent.

### 4.2 Gender differences

Behavioral results on accuracy show that when imposed with an object-based strategy, performances increase, regardless of the gender (seeFig. 5,Table 1). This goes in line with previous findings showing that using a spatial strategy to solve mental rotation is more effective (Halpern, 2013;Linn & Petersen, 1985;Shepard & Metzler, 1971). However, males still perform better than women in this condition. A possible explanation arises for this phenomenon when this behavioral result is linked with the functional data.

We found that gender affected the activity of specific brain regions. These findings are in line with previous electroencephalographic evidence that gender affects the temporal dynamics of the brain activity related to MR (Griksiene et al., 2019;Yu et al., 2009). When looking at the contrast OBS–control separated by gender, we observe that females activated an additional cluster in the somatosensory cortex (Fig. 8). This area is known to play an important role in processing tactile and proprioceptive information related to the body and its movements. Studies suggest that during MR tasks, the somatosensory cortex is involved in the simulation of body movements and the generation of a kinesthetic image of the body in space (de Lange et al., 2006;Maravita & Iriki, 2004;Parsons, 1994;Perruchoud et al., 2016). This process involves the recruitment of the body schema, which is a representation of the body’s posture and movements in space. The body schema is thought to be essential for MR because it enables individuals to simulate the movements of an imagined object or body part in space (Amorim et al., 2006). During MR tasks, the somatosensory cortex is more active in women than in men, suggesting that women may rely more on body-based processing during mental rotation (Hughes et al., 2012). When linked to the behavioral results, this led us to believe that this additional activation somehow affects the mental rotation process and interferes with the deployment of a pure spatial strategy. These findings hold particular significance due to the lack of discernible distinctions in the remaining conditions, whether pertaining to behavioral or functional aspects. It is noteworthy that the parity in performance between men and women in the alternate two conditions implies that gender-based variations in the mental rotation task likely stem, to some extent, from strategic factors. Consequently, the well-known gender disparities consistently identified within the domain might be diminished, as they would exclusively relate to scenarios wherein participants are compelled to adopt a visuospatial strategy.

### 4.3 Testosterone

This is even more interesting when the results of testosterone analyses are added to the equation. Some studies link testosterone levels to mental rotation performance (Burke et al., 2016;Hooven et al., 2004;J. Manning & Fink, 2018;Sadr et al., 2020). For example,J. Manning & Fink, 2018reports a negative correlation between 2D:4D and male performance, but not female performance. Nevertheless, neitherAustin et al. (2002), Bersier et al. (submitted), norCoolican and Peters (2003)discovered any such association in either sex, even with large population samples. In our study, when added as factors in an ANOVA, salivary testosterone did not help explain the difference in performance between genders. Quadratic regressions did not produce significant results, nor were Pearson correlations found with accuracy or reaction time (seeTable 2). Furthermore,Schöning et al. (2007)found no correlation between MR performance and hormonal data in men and women, but did observe region-specific correlations between testosterone and task-related activity in the left inferior parietal cortex. Interestingly, one of our regions of interest in our ROI analysis is very close to the region cited in Schöning et al. (2007), x = -44, y = -30, z = 40 vs. x = -48, y = -54, z = 34 for Schöning et al. (2007). Despite this proximity, we did not find any significant relationship in our analysis. This discrepancy might be attributed to differences in methodology, sample size, or specific aspects of task design and participant characteristics. Our findings, along with those of Schöning et al. (2007), highlight the complexity of the relationship between testosterone, brain activity, and cognitive performance, suggesting that further research is needed to unravel these intricate dynamics.

No effect was found for digit ratio in either in the ANOVAs or in the Pearson correlations. However, it is important to note that the lack of effect here may also be due to the fact that the D2:D4 digit ratio is simply not an appropriate proxy for prenatal testosterone levels, as warned in the meta-analysis bySorokowski and Kowal (2024).

### 4.4 Order of administration

A further finding lies in the training effect of the effector-based condition on the object-based condition. As shown inFigure 5, when participants perform the effector-based condition first (order A), their accuracy in the object-based condition that follows is significantly higher compared with the opposite order (order B). This can be interpreted as a facilitating effect due to the training through a motor strategy. Moreover, this enhancement in performance was present in men and women alike. Thus, the effects of motor strategy should not be ignored. Although it leads to lower performance in terms of accuracy in a mental rotation task, it seems to increase performance when it precedes the deployment of a spatial strategy. This accidental finding merits further investigation in future work.

## 5 Limitations

One limitation of this study is that the control condition was systematically performed at first. The motivation of this choice was to observe which strategy would be spontaneously used by the participants, in the absence of other indications, or any possible bias due to learning during the experiment.

The present study was designed to assess specifically the effect of testosterone on the brain activity related to MR. However, MR can be influenced by many other factors, including women’s sex hormones (Bernal & Paolieri, 2022;Bernal et al., 2020;Gurvich et al., 2023;Hausmann et al., 2000;Scheuringer & Pletzer, 2017). The present study focused the role of testosterone in mental rotation. Nevertheless, additional variations of other sex hormones, such as estradiol and progesterone, may play important roles in mental rotation. Investigating this topic could be the focus of future studies, which could recruit female participants during, for instance, early and late follicular phases, when estradiol and progesterone levels are different. Similarly, participants using oral contraceptives could be recruited during the inactive pill phase. This approach would help to recruit a more homogeneous group of women in terms of hormonal profiles and could provide more precise insights into the influence of sex hormones on mental rotation tasks.

It could be argued that participants may have started to mentally rotate the image before the cue. However, we would reject this hypothesis based on two main reasons. First, during a training session prior to the fMRI experiment, participants were explained and learned that they had to simply look at the target, without other mental activities, until the cue was presented. Second, to correctly accomplish the task, participants had to wait for the cue which would indicate the direction of MR, since the vertical could be reached by turning the image clockwise or counterclockwise. This direction was given by the cue and, if participants would begin MR before the cue, they may have faced the possibility of having to correct their mental process, which would cost a lot of mental energy and may have resulted in the activation of larger brain networks, which we did not detect

## 6 Conclusions

The present study investigated the effects of cognitive strategy and gender on MR-related brain activity. Our findings provide valuable insights into how different strategies and gender-specific factors influence both behavioral performance and underlying neural mechanisms during the mental rotation task. Behavioral results showed that men had higher accuracy in OBS than women. This difference was further elucidated by our fMRI data, which revealed that females showed additional activation in the somatosensory cortex in the OBS–control contrast, suggesting a reliance on body-based processing that may interfere with the deployment of spatial strategies. This finding suggests that gender differences in MR tasks are likely strategic rather than inherent, as performance was similar between genders in other conditions.

Importantly, we effectively demonstrated that different strategies, such as OBS and EBS, activate distinct brain regions and that these activations are associated with performance outcomes. In OBS, the pars opercularis—a region associated with spatial processing and mental rotation—was significantly activated. This activation correlated with higher accuracy for both groups, suggesting that OBS is an efficient spatial strategy. Conversely, EBS was associated with the activation of sensorimotor regions, reflecting the use of a motor strategy. However, EBS also resulted in a general lower accuracy at the behavioral level, suggesting that additional activation in sensorimotor regions may compromise the efficiency of MR.

Testosterone levels did not correlate with MR performance, challenging the notion that testosterone influences gender differences in spatial tasks. Finally, the order of administration showed that performing EBS first improved subsequent OBS performance for both groups, indicating a potential training effect from motor strategy to spatial strategy.

## Data Availability

Deidentified data and code are available on the github repository athttps://github.com/nadiaBRS/Mental-rotation-related-neural-interactions-between-gender-and-cognitive-strategy.
